# Multidimensional mechanisms of occupational stress among healthcare workers: a structural equation modeling analysis from the seventh Guangxi health service survey

**DOI:** 10.3389/fpsyg.2026.1781894

**Published:** 2026-04-22

**Authors:** Jinpei Li, Hongheng Li, Hongye Luo, Chunliu Lu

**Affiliations:** 1 School of Information and Management, Guangxi Medical University, Nanning, Guangxi, China; 2 Department of Operation and Management, Guangxi International Zhuang Medicine Hospital, Nanning, Guangxi, China

**Keywords:** ethnic minority areas in Western China, Guangxi Zhuang Autonomous Region, healthcare workers, structural equation model, work pressure

## Abstract

**Objective:**

The issue of work-related stress among medical personnel has become a key factor affecting the quality of healthcare services and the sustainable development of the medical industry. Taking healthcare workers in Guangxi as the research object, this study aims to systematically explore the multidimensional influencing factors and intrinsic action mechanisms of their occupational stress based on the structural equation model (SEM), clarify the direct and indirect effects among variables, and thus provide theoretical support and empirical evidence for formulating scientific and effective stress intervention strategies for Guangxi and other regions with similar characteristics.

**Method:**

Based on the dataset of medical staff in the Guangxi Zhuang Autonomous Region from the Seventh National Health Service Survey of China, 916 healthcare professionals from various medical institutions in Guangxi were selected as the research sample. A structured questionnaire was used to collect measurement data on latent variables including work environment, professional environment, job characteristics and personal characteristics, work experience, and work pressure. SPSS 26.0 was adopted for reliability analysis (Cronbach’s *α* coefficient) and exploratory factor analysis, while AMOS 26.0 was used to conduct confirmatory factor analysis for testing the structural validity of the scales, and construct a structural equation model to carry out path verification and effect analysis of the research hypotheses.

**Results:**

The model fitting results showed that all adaptation indices reached the ideal level (χ^2^/df = 4.001, GFI = 0.942, AGFI = 0.921, NFI = 0.943, CFI = 0.986, SRMR = 0.059, RMSEA = 0.057). Specific path analysis indicated that: (1) Work environment had a significant negative direct effect on work pressure (*β* = −0.272, *p* < 0.001), and also exerted a significant positive influence on job characteristics and personal characteristics (*β* = 0.454, *p* < 0.001) as well as work experience (*β* = 0.206, *p* < 0.001); (2) Professional environment had a direct negative impact on work pressure (*β* = −0.200, *p* < 0.001), and had significant positive effects on work environment (*β* = 0.561, *p* < 0.001) and work experience (*β* = 0.224, *p* < 0.001); (3) Work experience had a significant negative effect on work pressure (*β* = −0.115, *p* = 0.015), meaning poorer work experience led to higher occupational stress; (4) Job characteristics and personal traits had a significant positive direct effect on work pressure (*β* = 0.551, *p* < 0.001), which was contrary to the initial hypothesis, and they could indirectly affect work pressure through the mediating role of work experience (path effect value = 0.407, *p* < 0.001). In addition, the study verified a significant negative chain mediating effect of the path work environment→job characteristics and personal characteristics→work experience→work pressure (estimate = −0.021, 95% CI [−0.040,-0.006], *p* < 0.01). Work environment, professional environment, and work experience were the core factors for alleviating healthcare workers’ stress, acting on stress levels through both direct and mediating effects. All research hypotheses were supported except for the non-statistically significant path from professional environment to job and personal characteristics.

**Conclusion:**

In view of the characteristics of concentrated ethnic minorities and unbalanced urban–rural distribution of medical resources in Guangxi, efforts should be made to optimize the physical and social aspects of the work environment of medical staff, build a harmonious doctor-patient relationship to improve the professional environment, and enhance professional identity and career growth experience to strengthen work satisfaction. It is necessary to build a multi-stakeholder collaborative intervention system involving the government, medical institutions and society, implement resource inclination and differentiated policies for grassroots and ethnic minority medical and health institutions in Guangxi, and formulate targeted pressure reduction measures combined with regional and ethnic characteristics. This can provide practical guidance for alleviating the work pressure of healthcare workers in Guangxi, stabilizing the local medical talent team, and promoting the sustainable development of the medical and health industry in ethnic minority areas.

## 1 Background

The issue of poor physical and mental health among medical staff has been widely discussed in healthcare settings around the world. Even before the COVID-19 pandemic, the health of healthcare workers was already in crisis, manifesting in extremely high work pressure, burnout, mental health issues, and suicide rates (Melvin et al., 2025). For instance, an international study showed that 67% of healthcare workers experienced moderate to extremely high levels of psychological stress (Ghozy et al., 2022). Other studies have pointed out that compared to other healthcare workers, nurses faced a higher risk of negative psychological health outcomes (De Kock et al., 2021). Especially during public health emergencies, medical personnel faced even greater work pressure. For example, nurses, as one of the main forces in combating the COVID-19 pandemic, not only endured high-intensity and high-risk work but also faced immense physical and psychological stress (Chen et al., 2022). A study by Abdo et al. from Egypt showed that 66% of doctors and nurses in a tertiary hospital experienced moderate occupational burnout; meanwhile, research by Youssef et al. from Lebanon found that 90.7% of the study subjects exhibited moderate occupational burnout (Abdo et al., 2016; Youssef et al., 2022). Healthcare professionals worldwide have long faced issues such as work pressure and professional burnout, and public health emergencies have exacerbated this problem, as confirmed by several studies. So, what is the situation like in China? The following will focus on the situation in China.

In China, with the rapid development of the economy, the country’s main contradiction has shifted to the conflict between the growing needs of the people for a better life and the unbalanced and insufficient development. China’s healthcare system still cannot meet the health demands of the vast majority of the people, and the advancement of medical reform is aimed at addressing this issue. However, during the implementation of the reform, there is one particular group that requires more attention, and that is the healthcare workers. Healthcare workers are a very special group that plays a vital role in medical reform. Their special nature lies in two main aspects: on one hand, healthcare workers possess profound professional knowledge and skills. They know how to assess their own health and maintain their physical and mental well-being. On the other hand, due to the busy nature of their work, factors such as long hours and heavy workload can negatively impact their mental and physical health, leading to issues like depression, anxiety, and high work pressure.

The ‘China Practicing Physician Status White Paper’ (Chinese Medical Doctor Association, 2018) reveals that a mere 19.2% of physicians deem their health to be outstanding, 35% consider it mediocre, and 4.9% experience subpar health. Furthermore, healthcare professionals, in contrast to corporate staff, showed markedly greater psychological fatigue, suggesting that physicians endured considerable mental strain in their profession. Findings from the ‘2020 Survey on the Mental Health of Medical Workers’ (Chen and Wang, 2021) indicated that physicians rank highest in depression levels across various medical roles. Male healthcare professionals have a higher susceptibility to depression, with those aged 30–45 in different roles experiencing more depression compared to their counterparts in similar positions. In the context of burnout, the highest ratings are attributed to doctors, where female nurses outperform male nurses. Regarding psychological competencies, the distinction between male and female healthcare professionals is minimal, with the sole exception being the aspect of personal support. The ‘2021 China Physician Survey Report’ (Department of Sociology, Tsinghua University, 2021), authored by Tsinghua University’s Sociology Department, reveals that physicians dedicate, on average, 7.77 h daily to outpatient consultations, 1.47 h to research, and 5.77 days a week to work, signifying a remarkably high level of work intensity. Merely 11.86% of respondents in the survey feel they aren’t ‘fatigued,’ in contrast to the overwhelming majority of physicians who perceive research pressure as excessive, achieving 69.74%, Additionally, a majority of 55.56% perceived the stress from performance assessments to be overwhelming. Research indicates that from 2008 to 2018, China experienced 107 unexpected fatalities among its medical personnel, attributed to extreme work stress, with a mere 2.80% rate of rescue (Ce et al., 2020). Especially after the COVID-19 pandemic, apprehensions regarding the virus have escalated the physical and mental health challenges faced by healthcare professionals (Santana Lopez et al., 2022; Gao et al., 2021).

The aforementioned report and studies make it evident that the physical and mental well-being of medical personnel are a matter of concern and must not be ignored. Safeguarding the physical and mental well-being of medical staff, who are protectors of public health, forms the cornerstone of public health protection. As the population ages and medical personnel dwindle, the overall physical and mental well-being of healthcare workers becomes a societal challenge. The past few years have seen an increase in studies focusing on the mental well-being of healthcare professionals, especially concerning the elements that affect psychological stress and social backing. Enhancing the mental well-being of healthcare professionals has emerged as a trending subject (Mairiyemu et al., 2024).

The China Health Service Survey, as a core component of China’s national health statistics system, is conducted once every 5 years. It aims to systematically assess the health status of residents, the demands for and utilization of health services, and the working conditions of healthcare professionals. The survey employs a multi-stage stratified cluster random sampling method, with household interviews as the primary data collection approach. The survey provides key evidence for evaluating the effectiveness of health reforms, advancing the ‘Healthy China’ strategy, and formulating evidence-based policies. On September 6, 2023, the Guangxi Autonomous Region Health Commission held the 7th National Health Service Survey Training Workshop in Nanning, where survey work arrangements and related training were conducted. Therefore, this study focuses on healthcare professionals’ work pressure, using data from the National Health Service Survey to explore the factors that affect their work stress and propose targeted measures and recommendations.

Before conducting the research, it is necessary to define the variables.

Job characteristics and personal characteristics (JPC): refers to the work attributes perceived by medical professionals and their individual perceptions of work characteristics, including job significance, job demands, personal feelings toward work, sense of purpose, etc. They were measured by JPC2–JPC5 (JPC1 and JPC6 were excluded in exploratory factor analysis and confirmatory factor analysis and thus not included).

Work Environment (WE): refers to various resources provided by the workplace, such as work income, organizational respect, work equipment, etc. They were measured by WE2, WE4 and WE5 (WE1, WE3 and WE6 were excluded in exploratory factor analysis and confirmatory factor analysis and thus not included).

Work Experience (WEXP): refers to various perceptions during work, such as job satisfaction, perceived career growth, etc. They were measured by WEXP1–WEXP3 (WEXP4–WEXP6 were excluded in exploratory factor analysis and confirmatory factor analysis and thus not included).

Professional environment (PE): refers to the social environment affected by the quality of the doctor-patient relationship, such as doctor-patient relationship, social respect, etc. They were measured by PE1, PE3, PE4 and PE5 (PE2 and PE6 were excluded in exploratory factor analysis and confirmatory factor analysis and thus not included).

Work Pressure (WP): refers to work-related stress, job burnout, etc. They were measured by WP2–WP5 (WP1 and WP6 were excluded in exploratory factor analysis and confirmatory factor analysis and thus not included).

The analysis of influencing factors requires consideration of certain theories. This study is based on theories such as the stress cognitive appraisal theory and traditional stress theories.

Lazarus and Folkman’s cognitive appraisal theory of stress is alternatively referred to as the stress interaction model (Jiang and Wang, 2022). The theory serves to elucidate the mental evaluation and coping mechanisms individuals undergo in response to stress. The process of cognitive appraisal involves individuals evaluating how pertinent the environment is to their personal health. Negative outcomes will emerge if people believe that outside situations might be detrimental to their interests and they are unable to manage them effectively. On the other hand, if they are confident in their ability to surmount the obstacles, beneficial outcomes will arise. Cognitive appraisal theory of stress offers a comprehensive model for understanding individual cognitive responses to stressors, extensively applied in stress studies.

Conventional theories on stress research (Shi, 2002), exemplified by the models of Hendrix, Summers, Leap, and Steel, classify stress-inducing factors into three distinct categories: internal organizational elements, external organizational aspects, and personality characteristics. Factors within an organization are identified as the primary sources of occupational stress, primarily encompassing role conflict, unclear roles, excessive workload, time constraints, limited job independence, underutilization of skills, minimal involvement and control, managerial/supervisory dynamics, the organizational environment, and conflicts within the organization. The theory establishes connections between different workplace aspects, including the work setting, job features, individual characteristics, and perceptions of the job, and occupational stress. The study by Summers, DeCotiis, and DeNisi suggested a comparable framework, classifying work-related stress factors into four categories: personality attributes (like gender, dependent count), organizational structural features (formalization, centralization), organizational procedural features (including performance feedback, decision-making), and role attributes (such as role conflict, role ambiguity). According to the individual-environment fit theory (Sell et al., 1984), stress factors stem not solely from environmental or personal aspects, but also from the interplay between the individual and their surroundings. Lazarus’ theory of interaction suggests (Beehr, 2014) that: initially, in a given scenario, there is an interplay between the person and their surroundings; subsequently, the connection between the person and their surroundings transcends a mere blend of both, given the constant evolution of their relationship. Initially, it is crucial for a person to understand the close connection between their current job and their own job; subsequently, stress at work emerges only when job requirements surpass personal capabilities. The hypothesis associates personal traits with occupational stress. Pertinent studies indicate that the traits of a job are crucial in mitigating the adverse effects of technical stress on detrimental work conduct, which can be attributed to adverse outcomes stemming from subpar employee experiences (Li and Zhang, 2019). This further suggests that a combination of professional attributes and individual qualities can affect occupational stress and the overall work experience.

Additional theoretical studies emphasize the fundamental idea of the social information processing theory, indicating that employees’ views on job features are not completely impartial, but rather shaped by social signals like the attitudes of colleagues and the actions of leaders (Salancik and Pfeffer, 1978). Such social signals can be further classified within the workplace’s social milieu. Research conducted by Wang Wenxin et al. highlighted the influence of the doctor-patient psychological agreement on occupational stress and satisfaction. Diminishing stress and boosting job contentment can strengthen the psychological agreement between doctors and patients (Zheng et al., 2021), also classifiable within the professional sphere. Research conducted by Liu Xiaoxiao et al., likewise endorses this perspective (Liu et al., 2021).

**Figure 1:**
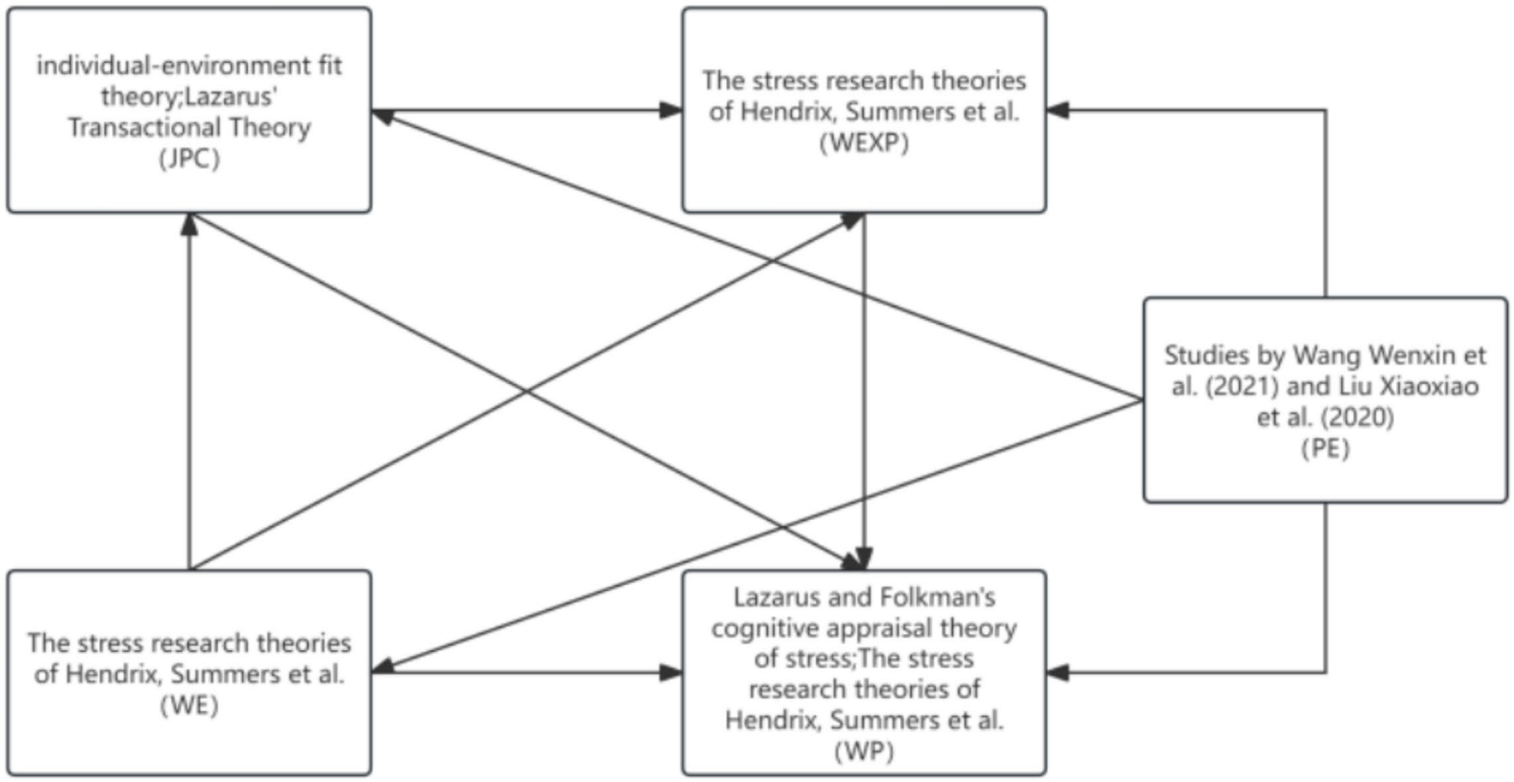
Theoretical integration model.

**Figure 2:**
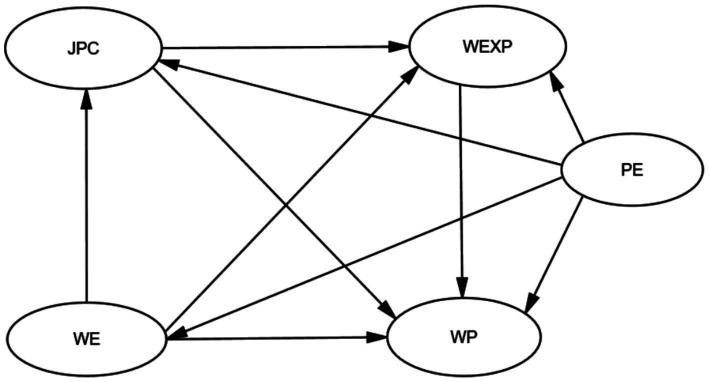
Hypothetical model.

Therefore, based on the above theoretical research, this study integrates these theories and simultaneously proposes the following hypotheses and models, as shown in Figures 1, 2.

A favorable doctor-patient interaction environment can promote the working environment of hospitals, and a sound interaction environment also indicates a desirable professional environment to a certain extent (Ansmann et al., 2014). Li pointed out in her study that a favorable professional environment provides medical staff with a good working environment (Li and Wu, 2018). On this basis, Hypothesis H1 is proposed.

*H*1: The professional environment has a significant positive impact on the work environment.

According to Edwards et al., person-environment fit occurs when there is good compatibility and adaptability between the environment and the individual, which leads to more positive work outcomes (Ye and Tang, 2025). Positive work outcomes may further contribute to better work experiences. On this basis, Hypotheses H2, H3, and H4 are proposed.

*H*2: The work environment has a significant positive impact on both job characteristics and personal characteristics.

*H*3: The professional environment has a significant positive impact on job characteristics and personal characteristics.

*H*4: Job characteristics and personal characteristics have a significant positive impact on work experience.

Work environment is directly correlated with job satisfaction, and a better work environment can promote job satisfaction (Lu et al., 2019), which constitutes part of work experience. Meanwhile, a better work environment can also facilitate personal development (Sun and Wang, 2017), which further influences work experience. On this basis, Hypothesis H5 is proposed.

*H*5: The work environment has a significant positive impact on work experience.

According to the previous study by Wang Wenxin and Liu Xiaoxiao, doctor-patient psychological contract shares the same definition as professional environment. The improvement of professional environment can alleviate job stress and enhance job satisfaction, which is in turn associated with work experience. On this basis, Hypothesis H6 is proposed.

*H*6: The professional environment has a significant positive impact on work experience.

According to Brummelhuis et al., a reduction in job resources can influence burnout and stress (ten Brummelhuis et al., 2011). Job resources generally include the work environment. According to Herzberg’s research, hygiene factors can alleviate the occurrence of burnout (Herzberg et al., 1959), which is consistent with the study by Li Wenzan showing that a poor work environment exerts negative impacts (Wei et al., 2025). On this basis, Hypothesis H7 is proposed.

*H*7: The work environment has a significant negative impact on work pressure.

According to a study by Li Wenzan, a favorable professional environment can alleviate stress among medical staff (Li et al., 2024). Meanwhile, a positive professional environment can improve the resilience of medical staff, thereby influencing burnout and further relieving stress (Wei et al., 2025). In addition, another study indicated that stress originating from patients is a major stressor (Choy and Wong, 2017). On this basis, Hypothesis H8 is proposed.

*H*8: The professional environment has a significant negative impact on work pressure.

According to Brummelhuis et al., an increase in job demands can affect burnout and stress (ten Brummelhuis et al., 2011). A study by Wang Wenzeng showed that self-determination (including competence, etc.) has a negative correlation with burnout and stress, and self-determination is similar to personal characteristics (Wang and Liu, 2020). On this basis, Hypothesis H9 is proposed.

*H*9: Job characteristics and personal characteristics have a significant negative impact on work pressure.

Studies have shown that job satisfaction (as part of work experience) has an impact on job stress: the higher the job satisfaction, the lower the job stress (Li and Zhang, 2019). Meanwhile, a meta-analysis by Carmen et al. (Quesada-Puga et al., 2024) indicated that the lower the satisfaction of ICU nurses, the more likely they are to suffer from problems such as burnout and excessive job stress.

*H*10: The work experience has a significant negative impact on work pressure.

## 2 Methods

### 2.1 Sources of data

The data of this study were derived from the dataset of medical staff in Guangxi Zhuang Autonomous Region of the 7th National Health Service Survey of China. The survey scope covered all tertiary general hospitals, some secondary hospitals in the sample counties (cities, districts) of Guangxi, as well as all community health service centers and township health centers in the sample townships (streets), with the involved counties (districts) including Binyang County of Nanning City, Liunan District of Liuzhou City, Hepu County of Beihai City, Gangbei District of Guigang City, and Luocheng Mulao Autonomous County of Hechi City; the survey targets were selected as clinical doctors, nursing staff, and preventive and health care personnel (including vaccination staff and health care personnel) from the aforementioned institutions. For the sampling of survey targets, in terms of hospitals, all tertiary general hospitals and some secondary hospitals in the sample counties (cities, districts) were included, with 20 clinical doctors and 10 nursing staff sampled from each tertiary general hospital; for community health service centers and township health centers, all such institutions in the sample streets and townships were covered, with 5 clinical doctors, 3 nursing staff, and 2 preventive and health care personnel sampled from each community health service center or township health center. If the number of staff in an institution was insufficient to meet the sample requirement, the survey was conducted based on the actual number of staff available. The principles for individual sample selection specified that all clinical departments within a hospital must be included in the sampling, and the sample should have a balanced distribution of professional titles, covering senior, intermediate, and junior professional titles. After preliminary screening, approximately 916 samples were included in this study. The analytical software used in this study were SPSS 26.0 and AMOS 26.0, with the structural equation model (SEM) adopted as the analytical method. The analytical steps included an analysis of the basic characteristics of the survey respondents, a common method bias test, reliability and validity analysis (including exploratory factor analysis, confirmatory factor analysis, convergent validity, and discriminant validity), and the construction of the structural equation model.

### 2.2 Material from the survey

Personal Information Form: It primarily encompasses details such as gender, age, marital status, highest educational attainment, professional technical designation, qualifications, workplace nature, work experience duration, and average monthly income. Average monthly earnings encompass salaries, bonuses, allowances, and various other income streams.

Job Characteristics and Personal Characteristics (JPC) serve as tools for assessing job attributes, including necessary skills, demands, and available resources. Xia and Lin (2013) along with individual traits during employment (such as having skills, a readiness to work, etc.). Six primary inquiries exist, with each question evaluated on a 4-point Likert scale from ‘Strongly Disagree’ to ‘Strongly Agree,’ scoring between 1 and 4; the Work Environment (WE) evaluates the work environment’s conditions, often encompassing physical and social elements. Comprising six primary queries, each item is evaluated on a 4-point Likert scale, spanning from ‘Strongly Disagree’ to ‘Strongly Agree,’ with ratings from 1 to 4; Work Experience (WEXP) quantifies the positive or negative emotions healthcare professionals encounter in their profession. The survey comprises six queries on a 6-point Likert scale, spanning from ‘Strongly Disagree’ to ‘Strongly Agree’ and scoring from 1 to 6, focusing on the Professional environment(PE) to assess the professional setting of healthcare workers, particularly regarding doctor-patient interactions and social factors. Six queries, evaluated on a 5-point Likert scale from ‘Very respectful’ to ‘Not respectful at all,’ ‘Very high’ to ‘Very low,’ ‘Very good’ to ‘Very poor,’ ‘Highly agree’ to ‘Highly disagree,’ and ‘Very satisfied’ to ‘Very dissatisfied,’ with ratings from 1 to 5; Work pressure (WP), primarily comprising six questions, gauges the stress experienced at work. Every item is evaluated on a 4-point Likert scale, spanning from ‘Strongly disagree’ to ‘Strongly agree,’ where the scores vary from 1 to 4. Detailed items are displayed in Table 1.

**Table 1:** Specific items in the questionnaire.

Survey items	Specific survey questions
JPC1	My work will have a significant impact on the lives or happiness of others.
JPC2	My job requires me to focus.
JPC3	The quality of my work will have an impact on many people.
JPC4	I think the job demands a lot from my abilities.
JPC5	My work is very meaningful and extremely important.
JPC6	When I get home, I can easily relax and put work aside.
WE1	The leaders and colleagues at my workplace have given me the respect I deserve.
WE2	Given the effort I have put in and the achievements I have already attained, I receive the respect and prestige I deserve in my work.
WE3	Considering the effort I have put in and my existing achievements, I have appropriate career prospects.
WE4	Given the effort I have put in and the achievements I have already attained, I have a fair salary.
WE5	The organization I work for provides the equipment and facilities that enable me to work efficiently.
WE6	I can easily access the various information I need for work at my workplace.
WEXP1	Work is a process of learning and growth for me.
WEXP2	Through work, my knowledge and skills are gradually improving.
WEXP3	In my work, I can try new things and actively tap into my potential.
WEXP4	I am satisfied with the benefits and compensation I receive at work.
WEXP5	I am satisfied with the training opportunities I have received at work.
WEXP6	Recently, I’ve been feeling very motivated to work hard.
PE1	The level of respect the patient shows toward you
PE2	The level of respect that society as a whole shows for your profession.
PE3	The level of trust the patient has in the services you provide
PE4	What do you think of the current doctor-patient relationship?
PE5	Do you feel that your work is recognized by the patients?
PE6	In most cases, patients will express appreciation for the services you provide.
WP1	In my work, I have to shoulder a lot of responsibilities.
WP2	Because of the heavy workload, I have always felt pressure on my time.
WP3	In recent years, my workload has been increasing steadily.
WP4	I easily get frustrated due to work-related stress.
WP5	People who know me say that I sacrifice too much for work.
WP6	I was still thinking about work when I went to bed.

## 3 Results

### 3.1 Fundamental details of the survey participants

Of the 916 medical professionals, 256 were male (27.9%) and 660 were female (72.1%), with an average age of 37.3 ± 8.6 years. Regarding marital status, 689 individuals are married, accounting for 75.2% of the total. In terms of professional credentials, 387 hold licenses as physicians (42.2%), 318 are registered nurses (34.7%), and 633 possess at least a bachelor’s degree (69.1%). Furthermore, 349 individuals possessed mid-level professional titles, accounting for 38.1%. In-depth details are presented in Table 2.

**Table 2:** Basic information table (*n* = 916).

Basic situation	Value
Gender [noun (%)]	
Man	256 (27.9)
Female	660 (72.1)
Age [M (P25, P75), years]	37 (30, 43)
Marital Status [Noun (%)]	
Not in marriage	214 (23.4)
Married	689 (75.2)
Other	13 (1.4)
County/District [n (%)]	
Hepu County, Beihai City	120 (13.1)
Gangbei District, Guigang City	257 (28.1)
Qixing District, Guilin City	29 (3.2)
Luocheng Mulao Autonomous County, Hechi City	158 (17.2)
Liunan District, Liuzhou City	192 (21.0)
Binyang County, Nanning City	160 (17.5)
Professional Qualification [Title (%)]	
Licensed physician	387 (42.2)
Practicing Assistant Physician	58 (6.3)
Traditional Chinese Medicine Practitioner	73 (8.0)
Traditional Chinese Medicine Practitioner Assistant	12 (1.3)
Registered Nurse	318 (34.7)
Other	68 (7.4)
Highest level of education [degree (%)]	
Doctoral graduate	3 (0.3)
Master’s graduate	33 (3.6)
Undergraduate degree	597 (65.2)
Junior College	246 (26.9)
Secondary vocational school/Secondary technical School	34 (4.0)
Professional technical title [Name (%)]	
Full Senior	15 (1.6)
Associate Senior	157 (17.1)
Intermediate	349 (38.1)
Junior (Level 2)	263 (28.7)
Junior (Level 1)	79 (8.6)
No professional title	53 (5.8)
Type of working organization [Noun (%)]	
Hospital	472 (51.6)
Community Health Service Center	187 (20.4)
Township health center	257 (28.1)
Years of work [M (P25, P75), years]	13 (8, 20)
Monthly average income [M (P25, P75), CNY]	5,500 (4,000, 7,000)

### 3.2 Standard technique for testing bias

Since data for multiple variables in this study were collected from the same respondents, common method bias may be a concern. Prior to formal hypothesis testing, Harman’s single-factor test was adopted to examine the severity of common method bias in this study. First, a CFA-based Harman’s single-factor test was performed, and when all items of the research variables were loaded onto one single common factor for model fitting, the model fit was extremely poor: χ^2^/df = 21.75, RMSEA = 0.15, CFI = 0.46, TLI = 0.42, SRMR = 0.14. Meanwhile, an EFA-based Harman’s single-factor analysis revealed that the percentage of total variance explained by a single factor was 31.4%, which is below 40%. These results indicated to some extent that common method bias in the present study was not severe (Zhang et al., 2020).

### 3.3 Analysis of reliability and validity

#### 3.3.1 Analysis of reliability

When performing a reliability analysis on each dimensions’ items, typically, a score above 0.8 signifies high internal consistency, whereas a score between 0.6 and 0.8 denotes high consistency (Jiang et al., 2010). The reliability of each dimension exceeded 0.700, indicating strong internal consistency across all dimensions, as shown in Table 3.

**Table 3:** Reliability analysis table (*n* = 916).

Latent variable	Item code	Number of questions	Cronbac’s α
JPC	JPC1-JPC6	6	0.745
WE	WE1-WE6	6	0.870
WEXP	WEXP1-WEXP6	6	0.875
PE	PE1-PE6	6	0.866
WP	WP1-WP6	6	0.829

#### 3.3.2 Analysis of structural validity

##### 3.3.2.1 Exploratory factor analysis

The extraction of factors was performed through the principal component technique. The derivation relied on the scree plot test, Kaiser’s eigenvalue criterion for eigenvalues above 1, and the rule that the total variance percentage must surpass 60%. Five distinct factors were identified in total. Principal axis factoring was the chosen extraction technique, while the oblique method served as the rotation method. Values below 0.4 were omitted to maintain a distinct factor configuration in the factor loading chart. Hair states that factor loadings ranging from 0.30 to 0.40 are deemed the lowest permissible threshold, and values exceeding 0.50 are typically seen as noteworthy (Hair et al., 2013). In the end, five items—JPC6, WP1, WEXP4, WEXP5, and WEXP6—were excluded, with the outcomes displayed in Table 4.

**Table 4:** EFA factor loadings table.

Item	Factor				
	1	2	3	4	5
JPC1			0.484		
JPC2			0.694		
JPC3			0.821		
JPC4			0.821		
JPC5			0.710		
WP2				0.707	
WP3				0.876	
WP4				0.698	
WP5				0.702	
WP6				0.657	
WE1		0.473			
WE2		0.726			
WE3		0.812			
WE4		0.729			
WE5		0.702			
WE6		0.722			
WEXP1					0.892
WEXP2					0.983
WEXP3					0.763
PE1	0.839				
PE2	0.705				
PE3	0.843				
PE4	0.677				
PE5	0.751				
PE6	0.546				

##### 3.3.2.2 Confirmatory factor analysis

**Figure 3:**
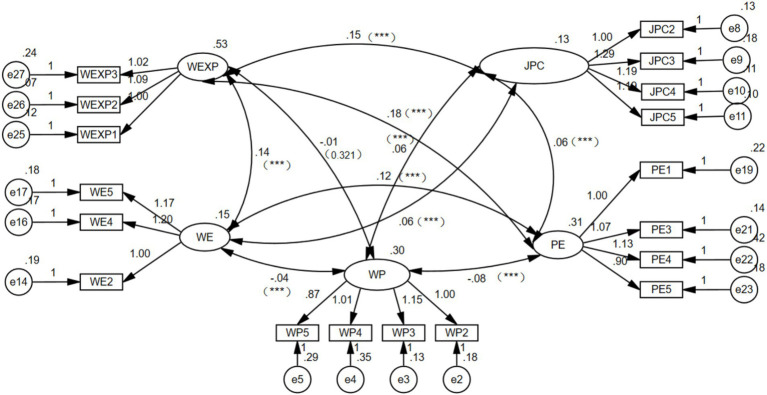
CFA model.

The creation of a confirmatory factor model led to the exclusion of items with loadings under 0.5, resulting in the final removal of WE1, WE6, WE3, PE2, WP6, JPC1, and PE6 (Wu, 2013). Consequently, as depicted in Figure 3, the model attained the optimal fit, evidenced by χ^2^/df = 4.019, GFI = 0.942, AGFI = 0.921, NFI = 0.943, CFI = 0.956, SRMR = 0.059, RMSEA = 0.057.

##### 3.3.2.3 Analyzing convergent and discriminant validity

Table 5 displays the analysis of convergent validity. Should the square root of the AVE (Average Variance Extracted) for two constructs exceed their correlation coefficient (or if both constructs’ AVE values surpass the squared correlation coefficient), it signifies the constructs’ strong discriminant validity. The foundation of this theory lies in the notion that the variance accounted for by the elements (measurement variables) of a latent construct ought to surpass that accounted for by another latent construct (Wu, 2013). Every AVE value exceeds 0.5, and the diagonal holds the square roots of these AVE values, each of which exceeded the absolute values of the corresponding correlation coefficients in the matrix, indicating strong discriminant validity among the constructs.

**Table 5:** Convergent validity.

Latent variable	CR	AVE	Latent variable				
			WP	JPC	WE	PE	WEXP
WP	0.840	0.570	0.755				
JPC	0.847	0.581	0.306***	0.762			
WE	0.756	0.508	−0.196***	0.446***	0.713		
PE	0.854	0.594	0.249***	0.291***	0.554***	0.771	
WEXP	0.925	0.806	−0.036	0.563***	0.510***	0.454***	0.898

***indicates *p* <0.001.

Table 6 displays the analysis of discriminant validity. HTMT represents the ratio of between-trait correlation to within-trait correlation. This denotes the average correlation among indicators of various constructs in comparison to the average correlation within indicators of a single construct. As per the standards established in scholarly works (Henseler et al., 2015), the HTMT figure ought to remain below 0.85. In the present model, all HTMT values were below 0.85, confirming strong discriminant validity among the study variables.

**Table 6:** Analysis of discriminant validity.

Dimension	WP	JPC	WE	PE	WEXP
WP					
JPC	0.311				
WE	0.194	0.441			
PE	0.283	0.274	0.568		
WEXP	0.057	0.566	0.529	0.472	

### 3.4 Modeling of structural equations

**Figure 4:**
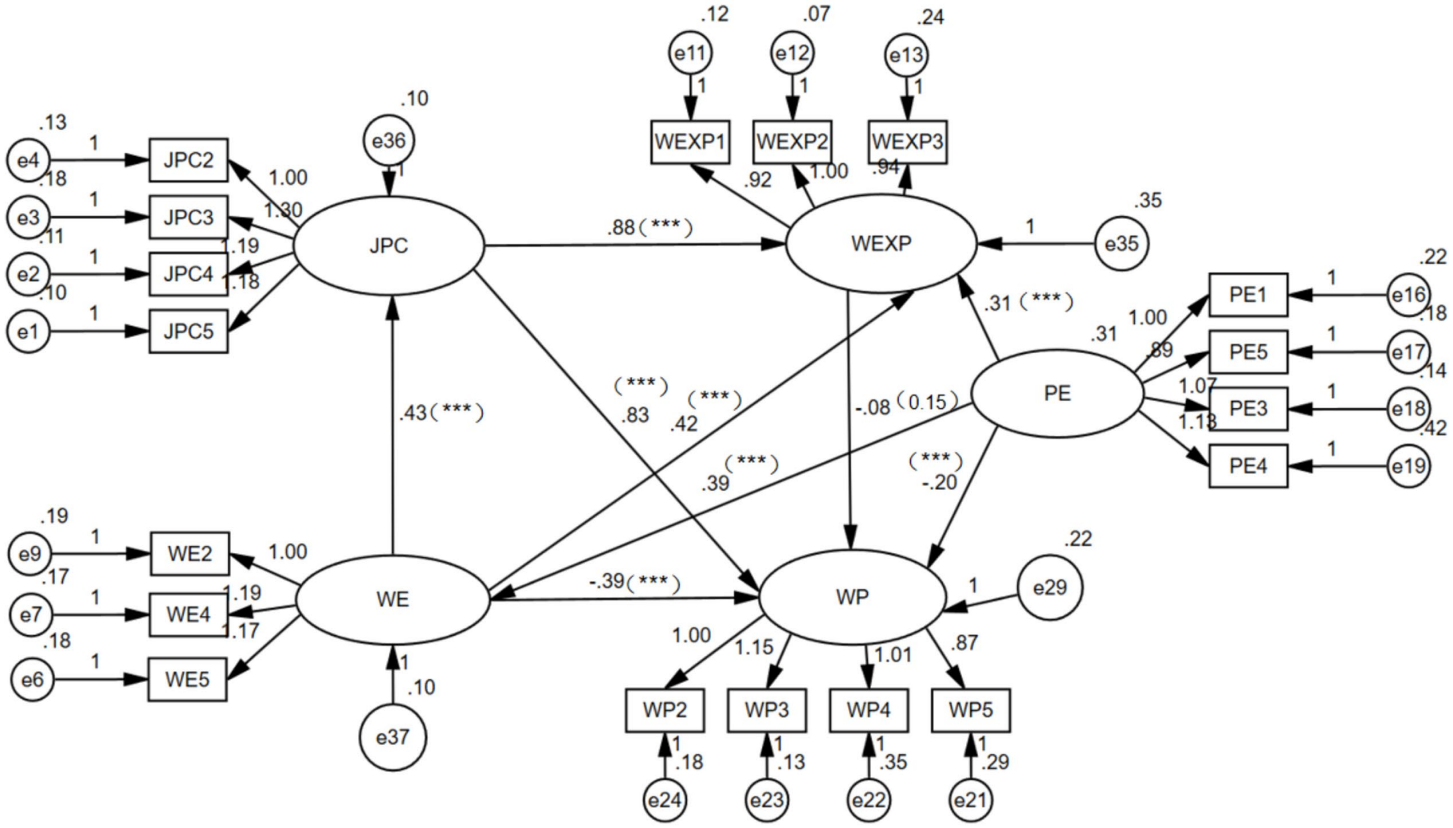
Revised structural equation model.

Following the research hypotheses, a structural equation model was developed, and the inconsequential link from H3; professional environment to job and personal traits (*p* = 0.178) was eliminated. Table 7 presents the model fit indices, all of which met the recommended criteria, indicating a good model fit (Wu, 2013). Figure 4 displays the ultimate model, which is derived from the altered version.

**Table 7:** Fitting results.

Fit index	x^2^/df	GFI	AGFI	NFI	CFI	SRMR	RMSEA
Suggested value	<5	>0.9	>0.9	>0.9	>0.9	<0.08	<0.08
Actual value	4.001	0.942	0.921	0.943	0.986	0.059	0.057

The results of the path analysis and hypothesis testing are presented in Table 8. All path coefficients were statistically significant (*p* < 0.05), and all research hypotheses were supported except for H9. The effect values of work pressure are shown in Table 9. Meanwhile, the serial mediation model consisting of WE → JPC → WEXP → WP was significant, indicating that work environment can exert a negative influence on work pressure through job characteristics, personal characteristics, and work experience, as shown in Table 10.

**Table 8:** Path analysis and hypothesis results.

Hypothesis	Path	Standardized Estimate	Estimate	S. E	C. R	*p*	Supported/not supported
H1	PE→WE	0.561	0.385	0.031	12.387	***	Support
H2	WE→JPC	0.454	0.429	0.042	10.125	***	Support
H4	JPC→WEXP	0.407	0.881	0.082	10.795	***	Support
H5	WE→WEXP	0.206	0.421	0.096	4.409	***	Support
H6	PE→WEXP	0.224	0.314	0.055	5.7	***	Support
H7	WE→WP	−0.272	−0.387	0.081	−4.746	***	Support
H8	PE→WP	−0.200	−0.196	0.046	−4.239	***	Support
H9	JPC→WP	0.551	0.831	0.079	10.518	***	Not supported
H10	WEXP→WP	−0.115	−0.08	0.033	−2.433	0.015	Support

P represents significance, and *** indicates *p* < 0.001. Typically, when a two-tailed test shows *p* < 0.05, it means the path coefficient estimate is significantly different from 0, indicating that the unstandardized path coefficient is significant.

**Table 9:** Effect values of influencing factors on work pressure.

Dimension	WP		
	Standardized total effect (β)	Standardized direct effect (β)	Standardized indirect effect (β)
PE	−0.263	−0.200	−0.063
WE	−0.066	−0.272	0.205
JPC	0.504	0.551	−0.047
WEXP	−0.115	−0.115	0.000

**Table 10:** Chain mediation of work pressure.

Type of mediating effect	Estimate	95 CI lower bound	95% CI upper bound	*p*-value	Significance
WE→JPC → WEXP→WP	−0.021	−0.040	−0.006	0.008	*p* < 0.01

## 4 Discussion

### 4.1 The influence of the work environment

The standardized coefficient of work environment on work pressure was −0.272, with a C. R. value of −4.746 and *p* < 0.001, indicating that each one-standard-deviation increase in work environment was associated with a 0.272-standard-deviation decrease in work pressure. Therefore, work environment had a significant negative effect on work pressure, meaning that the better the work environment, the lower the work pressure. This is consistent with the research by Mei Songli, in which Mei argued that both the physical environment and the social environment are negatively correlated with the occupational burnout of medical staff (Mei et al., 2023). Put differently, an improved work environment leads to reduced occupational stress. Likewise, the professional environment positively influences both job characteristics and personal characteristics. An optimal work environment fosters a positive workplace atmosphere, impacting the workplace and facilitating the alignment with personal characteristics, thereby boosting individual work readiness. The quality of the workplace positively influences the work experience; an improved environment leads to an enhanced personal experience. The aforementioned findings further demonstrate the impact of the work environment’s quality on all facets of the job. The workplace environment includes aspects of both social and physical surroundings. The term ‘social environment’ primarily denotes the interpersonal dynamics among healthcare professionals, their superiors, or peers, along with mental support, among other aspects. The term ‘physical environment’ denotes the exposure hazards encountered by medical personnel, which are inherent to their profession. This environment, coupled with diverse material supports, can additionally mitigate risks (Mei et al., 2023). Healthcare institutions can adopt two main strategies: first, improving the physical working conditions of medical staff. Healthcare facilities ought to offer top-tier fundamental facilities to the greatest extent feasible, ensuring that the working environment is safe, standardized, and hygienic, including the modernization of medical devices, office computers, and efficient, smart work networks. Additionally, it is crucial for leaders to focus on the health and physical well-being of healthcare professionals, enhance their leadership approaches, foster a unified and integrated medical team, and conduct daily tasks to fortify interdepartmental links, thereby establishing an effective communication environment. Recent research has incorporated the idea of a “magnetic nursing workplace,” demonstrating its effectiveness in enhancing the work setting and boosting nurse job contentment (Wang et al., 2019). Prominent healthcare organizations can utilize and implement this framework to enhance their professional setting.

At the governmental level, investment in the medical field should be increased, policies guaranteeing a better work environment should be improved, supervision over environmental improvement in medical institutions should be strengthened, and the effective implementation of relevant policies should be promoted. At the departmental level, department managers should base their management on actual working conditions, optimize the physical layout of departments, establish internal communication platforms, respond to the demands of medical staff in a timely manner, and create a mutually supportive and collaborative atmosphere. Meanwhile, differentiated measures should be taken for medical staff in different positions: for nurses with a heavy workload, increasing staffing is recommended; for doctors under research pressure, optimizing the assessment and reward mechanisms is suggested.

### 4.2 The influence of the professional environment

The standardized coefficient of professional environment on work pressure was −0.200, with a C. R. value of −4.239 and *p* < 0.001. These results indicate that each one-standard-deviation increase in professional environment was associated with a 0.200-standard-deviation decrease in work pressure. Professional environment thus had a significant negative effect on work pressure, meaning that a better professional environment was related to lower work pressure, which is consistent with the findings of Thomas and Liu Xiaoxiao. In their study, Liu Xiaoxiao found that perceived doctor-patient relationship exerted a significant negative predictive effect on work-related stress—specifically, the poorer the perceived doctor-patient relationship, the higher the level of work-related stress (Liu et al., 2021; Thomas et al., 2007). Put differently, an improved practice setting leads to reduced stress at work. Additionally, the working environment positively influences both the workplace and the overall work experience.

Multiple elements play a role in shaping the professional environment. Bridging the professional divide between healthcare professionals and patients is challenging, given that patients often find it hard to grasp medical treatments because of their limited knowledge. Moreover, false information or poor social media interaction can deceive certain patients into questioning healthcare providers, leading to intensifying disputes between doctors and patients. Such circumstances may result in medical conflicts, harmful assaults on healthcare professionals, and various other negative events.

Enhancing the professional environment necessitates joint endeavors. By boosting healthcare funding, broadening insurance to alleviate health-related financial strains, advocating for medical liability insurance and external mediators to alleviate doctor-patient conflicts, and curtailing the profit-centric approach of healthcare facilities, the government can also improve their public welfare operations. Healthcare facilities ought to enhance their service procedures, streamline the process of registration and prescriptions, bolster the training and education in medical ethics and doctor-patient communication, and motivate healthcare professionals to embrace empathetic approaches. It is essential for society to utilize media’s beneficial impact to deliver unbiased coverage of the healthcare sector’s present circumstances. A multi-stakeholder collaboration is needed to improve the professional environment, thereby reducing the workload of medical staff.

Medical departments can improve the on-site response plans for doctor-patient conflicts, strengthen training for medical staff in emergency communication and self-protection, and resolve minor disputes in a timely manner. In addition, departments can optimize the diagnosis and treatment layout and workflow, reduce repetitive tasks, and improve work efficiency and professional comfort.

### 4.3 The effects of work experience

The standardized coefficient of work experience on work pressure was −0.115, with a C. R. value of −2.433 and *p* < 0.05, indicating that each one-standard-deviation increase in work experience was associated with a 0.115-standard-deviation decrease in work pressure. Work experience thus had a significant negative effect on work pressure, meaning that the better the work experience, the lower the work pressure. This is similar to a study conducted by Thorsteinsson, which revealed that factors such as perceived competence and workplace support exert an indirect influence on stress-related health outcomes, such as depression (Thorsteinsson et al., 2014). Put differently, improved job satisfaction correlates with reduced work-related stress. Job satisfaction can be categorized into beneficial and detrimental factors. Enhanced job contentment, like increased job fulfillment or peaceful interactions with coworkers, can reduce stress. On the other hand, diminished job contentment, marked by heightened sensations of injustice, extreme tiredness, juggling multiple tasks, or ambiguous duties, may escalate occupational stress.

At the governmental level, efforts should be made to improve the salary distribution mechanism for medical staff, balancing fairness and efficiency. Salaries should be clearly linked to work performance and professional competence to reduce negative perceptions caused by unfair compensation. Meanwhile, labor protection policies should be implemented to eliminate unpaid overtime and overwork, and effectively protect the legitimate rights and interests of medical staff, and enhance their positive work experience.

At the hospital level, job responsibilities for each position should be sorted out and work boundaries clarified to avoid overlapping tasks and ambiguous duties, thus reducing internal work friction for medical staff. A sound career development platform and promotion pathway should be established to strengthen medical staff’s professional identity and sense of accomplishment, so as to improve work experience and alleviate work pressure.

At the departmental level, shifts should be arranged scientifically and rationally according to workload, with clear job division and reduced repetitive tasks to prevent overwork among medical staff. A mutually supportive and collaborative departmental atmosphere should be created to strengthen workplace support for frontline medical staff, effectively improve their positive work experience and relieve work pressure.

### 4.4 The impact of job characteristics and personal characteristics

The standardized coefficient of job characteristics and personal characteristics on work pressure was 0.551, with a C. R. value of 10.518 and *p* < 0.001, indicating that each one-standard-deviation increase in job characteristics and personal characteristics was associated with a 0.551-standard-deviation increase in work pressure. Job characteristics and personal characteristics had a significant positive effect on work pressure, and this path was not consistent with the original hypothesis. In contrast, job characteristics and personal characteristics exerted a positive effect on work experience, which was generally consistent with the study by Li and Zhang (2019). Although a high sense of work meaning endowed individuals with a strong sense of value and mission, stimulated their internal work motivation, improved work engagement and initiative, and made them more willing to strive for goals, it also strengthened their awareness of responsibilities. This encouraged them to take on more duties and raised self-requirements, thus bringing extra psychological pressure and burden. Such strong identification with work value made people care more about performance and responsibility fulfillment, easily leading to long-term mental tension and showing the dual effect of boosting motivation while increasing pressure. While job and personal characteristics do not have a direct negative impact on work pressure, they can indirectly influence work experience, thereby acting as a mediator in work pressure. Improving job and personal characteristics requires efforts in two key aspects. Initially, regarding occupational traits: it is essential for hospitals to define the job requirements and available resources for healthcare personnel in their roles, fine-tune the management of work in response to these resource demands, and address issues related to the job. Concurrently, assignments ought to be distributed according to the unique traits of each healthcare professional, and the training must be intensified to boost their abilities, thus enriching their professional experience. Secondly, individual traits: healthcare professionals can concentrate on enhancing their own expertise and abilities to adjust to a more intricate professional setting. As an illustration, the application of artificial intelligence models aids in diagnosing and managing everyday activities, facilitating their work management and enhancing their individual work experiences.

The government should improve the policies for resource allocation and professional environment protection in grassroots medical care, and increase investment in human resources, equipment and information technology support for grassroots medical institutions, so as to optimize the job characteristics of medical staff at a macro level and reduce work pressure caused by the external environment. Departments can implement a refined task allocation and skill support mechanism matching personal characteristics, carry out targeted professional training, optimize frontline workflow, and indirectly alleviate work pressure by improving the work experience of medical staff.

### 4.5 Discussion and suggestions on the chain mediation

This study confirmed a significant negative chain mediating effect of work environment → job and personal characteristics → work experience → work pressure (estimate = −0.021, 95% CI [−0.040, −0.006], *p* < 0.01), which is also an indirect pathway through which work environment alleviates work pressure among medical staff. This transmission process is consistent with the cognitive appraisal theory of stress and the person-environment fit theory. A favorable work environment can positively shape adaptive job and personal characteristics, and job characteristics matching individual traits can further enhance medical staff’s positive work experience in learning, growth and skill improvement. Such positive work experience can in turn directly reduce work pressure. This reveals the layered internal mechanism among multiple variables and provides a progressive intervention approach to relieve work pressure for medical staff. That is, starting from optimizing the work environment as the source, promoting the precise matching of job characteristics and personal characteristics, and ultimately forming a closed loop by enhancing positive work experience, so that the effect of work environment optimization can be transmitted through multiple links to achieve pressure relief.

Based on this mechanism, interventions can be carried out in three aspects: first, the government and medical institutions should jointly optimize the work environment at the source. For county-level and grassroots medical units in Guangxi, hardware facilities should be improved, salary incentives and organizational respect should be implemented to lay a foundation for characteristic matching. Second, medical institutions should promote the precise fit between job characteristics and personal characteristics, carry out refined task allocation and targeted skill training according to medical staff’s professional ability and work willingness, and shape job characteristics consistent with individual traits. Third, all medical departments should strengthen the cultivation of positive work experience, build career development platforms, create a mutually supportive and collaborative atmosphere, and continuously improve medical staff’s experience of learning and growth.

### 4.6 Discussion and suggestions on alleviating work pressure of medical staff in Guangxi

Combined with the actual situation of the survey in Guangxi, based on the characteristics of concentrated ethnic minorities in western Guangxi and the uneven urban–rural distribution of medical resources, this section puts forward strategies from three aspects: government, medical institutions and society, focusing on the four core influencing factors of work environment, professional environment, work experience, and job and personal characteristics, to alleviate the work pressure of medical staff at different levels in Guangxi.

At the governmental level, emphasis should be placed on resource guarantee and policy adaptation, with a focus on tilting toward grassroots and ethnic minority counties and districts. First, increase capital investment in county-level grassroots medical institutions, improve physical environments such as diagnosis and treatment equipment and intelligent work networks, add post allowances for medical staff in remote counties and districts, and ensure that salaries match their efforts. Second, fully promote medical liability insurance in grassroots counties and districts, establish a third-party mediation mechanism for doctor-patient conflicts that includes mediators from the Zhuang and Mulao ethnic groups, and carry out positive publicity on the medical industry through local media to enhance social respect for medical staff. Third, implement differentiated assessment, reduce the scientific research assessment indicators for grassroots medical staff, and focus assessments on clinical service performance, and build a clinical skills training platform linking urban and grassroots areas.

At the medical institution level, measures should be taken based on their own positioning to help medical staff alleviate excessive pressure. For example, in urban hospitals, optimize the weight of clinical and scientific research assessment, increase staffing for nursing positions, and promote the “magnetic nursing workplace” model; in grassroots medical institutions, simplify high-frequency work processes such as vaccination and health record management, and carry out special training on doctor-patient communication; all medical institutions need to clarify the work boundaries of each position to avoid redundant tasks, and optimize task allocation based on medical staff’s professional skills and personal strengths, and build clear career promotion channels to enhance their professional identity.

At the social level, rely on Guangxi’s ethnic characteristics to create a positive supportive atmosphere and improve the external professional environment. Give play to the cultural advantage of mutual assistance among ethnic minorities, carry out community medical and nursing care activities in grassroots areas to solve practical problems such as medical staff’s children’s care and commuting; local media should abandon the tendency of over-exaggerating medical disputes, objectively report the work contributions of medical staff, and strengthen the popularization of medical knowledge to reduce doctor-patient conflicts caused by professional cognitive deviations; encourage public welfare organizations to provide free psychological evaluation and counseling services for medical staff to alleviate their psychological pressure.

In summary, the government, medical institutions and society need to closely focus on the regional and ethnic characteristics of Guangxi, implement targeted pressure reduction measures in Guangxi’s medical practice, and establish a long-term multi-stakeholder collaboration mechanism, effectively alleviate the work pressure of medical staff, and stabilize the medical talent team in Guangxi.

## 5 Overview

This study explored how the work environment, professional environment, work experience, job characteristics, and personal characteristics influence work pressure, as well as their interrelationships. The results indicated that the work environment, professional environment, and work experience significantly reduced work-related stress. A diverse strategy is essential to reduce the stress experienced by healthcare professionals for thorough enhancement.

This study systematically reveals the influencing pathways of work pressure among medical staff, with a rigorous design and regionally representative sample, providing valuable empirical evidence for local health policy and practice. Several aspects can be further explored in future research: First, the cross-sectional design effectively captures the correlational patterns among variables. Due to the nature of this design, causal inference may benefit from additional longitudinal evidence. Longitudinal studies can be conducted to further validate causal relationships. Second, the sample was focused on Guangxi, which well reflects the characteristics of the southwest region and carries strong practical implications. To enhance generalizability at the national level, future studies may adopt a multi-center, cross-regional sampling strategy. Third, standardized scales were applied to ensure data consistency and reliability. Incorporating objective indicators or third-party assessments in future work could further enrich measurement dimensions and strengthen comprehensiveness. Fourth, path analysis clearly identified the core influencing pathways and laid a solid foundation for model construction. Future research may introduce mediating and moderating effects to explore the mechanisms of work pressure in greater depth.

Future improvements will be made in the following aspects: First, a longitudinal follow-up design will be adopted in subsequent studies to further verify the causal relationships and temporal order among variables; additionally, more comprehensive measurement methods combining subjective questionnaires with objective indicators or third-party evaluations will be applied to improve data reliability; finally, mediating and moderating effects will be integrated into the analysis, and variables such as organizational support will be introduced as moderators to construct a more robust and applicable model for the influencing factors of medical staff’s work pressure.

## Data Availability

The data analyzed in this study is subject to the following licenses/restrictions: this dataset is derived from the Guangxi survey of the China Health Services Survey. Currently, the corresponding report has not yet been completed, so this dataset is not available for public access at this time. Requests to access these datasets should be directed to Hongye Luo, Hongye283@163.com.
